# Naloxone Prescribing Among Long-Term Opioid-Prescribed Patients: Disparities and Opportunities

**DOI:** 10.7759/cureus.82180

**Published:** 2025-04-13

**Authors:** Brian Yorkgitis, Ira Harmon, Azad Khan, Fern Webb, Gabriel Brat

**Affiliations:** 1 Surgery, Indiana University School of Medicine, Indianapolis, USA; 2 Center for Data Solutions, University of Florida College of Medicine, Jacksonville, USA; 3 Surgery, Beth Israel Deaconess Medical Center, Boston, USA; 4 Biomedical Informatics, Harvard Medical School, Boston, USA; 5 Center for Health Equity and Engagement Research, University of Florida College of Medicine, Jacksonville, USA

**Keywords:** naloxone, opioid-induced respiratory depression, opioid prescribing, opioids, overdose

## Abstract

Introduction: Opioids have a risk for opioid-induced respiratory depression (OIRD) that can be fatal. Naloxone has been proven to reverse opioid effects. However, co-prescribing of naloxone with opioids is underutilized. Through the query of a national outpatient healthcare dataset, the study aims to discern differences in co-prescribing naloxone to provide a framework of education to formulate recommendations on naloxone prescribing.

Methods: A retrospective review of a de-identified, national outpatient healthcare dataset was analyzed for patients with a pain-provoking condition and receipt of ≥3 opioid prescriptions. Demographics, medical history, and prescribing data were used to identify high-risk patients for OIRD along with co-prescribing of naloxone between 2015 and 2021 and analysis between 2022 and 2024.

Results: Among 181,964 patients, 1807 (1%) received a naloxone prescription of the total cohort. Examining co-prescribing for high-risk patients only, 107 (3.3%) were receiving >50 MME/day opioids, 468 (2.6%) were concomitantly prescribed benzodiazepines, and 273 (7.8%) who had opioid use disorder (OUD) history received naloxone prescriptions. Upon logistic regression, the likelihood of naloxone co-prescribing among patients with a history of OUD showed an odds ratio (OR) of 6.63 (95% CI 5.76-7.63; p>0.001), and that among patients concomitantly prescribed benzodiazepines showed an OR of 2.76 (95% CI 2.47-3.09; p>0.001). Hispanic patients (OR 0.87; 95% CI 0.76-0.98; p=0.27) and those uninsured or with unknown insurance (OR 0.65; 95% CI 0.51-0.81; p<0.001) were less likely to receive a naloxone prescription. Black (OR 1.30; 95% CI 1.15-1.47; p>0.001) and unknown race (OR 1.38; 95% CI 1.15-1.66; p=0.001) patients were more likely to receive naloxone prescriptions.

Conclusion: Despite recommendations that high-risk opioid-prescribed patients receive naloxone prescriptions, only a fraction are in receipt. There is variation among patient populations in co-prescribing, leaving opportunities to improve universal precautions that include naloxone co-prescribing to all high-risk patients for OIRD.

## Introduction

Opioids are often prescribed for pain conditions. These medications in pain management must be prescribed with caution given their risks for opioid-induced respiratory depression (OIRD) that can lead to death. Risk factor mitigation for OIRD is multifaceted including the identification of conditions and medications that can increase risk. These include a history of opioid use disorder (OUD), taking >50 morphine milligram equivalents (MME) per day, and concomitant benzodiazepine use [1,2].

Naloxone is an opioid receptor antagonist that reverses the effect of opioid overdose, including OIRD [3]. Before 2015, naloxone was available only in injectable form, limiting its accessibility for non-medical professionals. The Food and Drug Administration (FDA) approved nasal naloxone for prescription dispensing to patients, recognizing its efficacy to save lives and ease of use. As a powerful tool in reducing opioid-related deaths, it is available over-the-counter (OTC) to increase availability [4].

Prescribers of opioids should identify patients with risk factors for opioid-related adverse events, including the aforementioned risk factors. The Centers for Disease Control and Prevention (CDC) included offering naloxone and overdose prevention education to both patients and their household members as part of the CDC's Guideline for Prescribing Opioids for Chronic Pain and other opioid safety campaigns [5-7].

A wide variation in prescribing naloxone for high-risk patients has been described [8]. In 2019, the CDC has reported that primary care clinicians only write 1.5 naloxone prescriptions per 100 high-dose opioid prescriptions [1]. Efforts to increase co-prescribing with state mandates have led to a more recent increase in this practice [9]. As with opioid prescribing, disparities extend to prescribing life-saving adjuncts. One study examining patients with fracture or chronic pain syndromes showed patients of color were less likely to receive opioid analgesic prescriptions and naloxone prescriptions than their non-Hispanic White contemporaries [10]. In a Veterans Administration study, differences in naloxone prescribing were identified with female, Asian, Black, and Hispanic patients less likely to receive a prescription [11]. 

Given the significant public health benefit naloxone possesses to assist in decreasing ORID and death, identifying patients who can benefit from co-prescribing is paramount. Through examination of a national community healthcare clinic database among long-term opioid patients, we sought to identify differences in naloxone prescribing among different populations and high-risk factors to identify opportunities to improve co-prescribing practice. 

## Materials and methods

This retrospective study analyzed patients from community-based health centers included in OCHIN Accelerating Data Value Across a National Community Health Center Network (ADVANCE) Epic (Epic Systems Corporation, Madison, Wisconsin, United States) electronic health record (EHR) system. OCHIN is a national nonprofit health IT consultancy that provides a fully hosted and shared EHR platform to over six million patients across more than 1,600 clinics in 33 states [12].

The study cohort consisted of de-identified patient encounters between January 1, 2015, and December 31, 2021, with analysis between 2022 and 2024. Those who were 18 years or older at the time of the encounter with three or more opioid prescriptions and a diagnosis of at least one pain-provoking condition of interest were included. Patients were categorized into seven categories of pain-provoking conditions using International Classification of Diseases (ICD) 9 and 10 with the assistance of previous pain condition work done using OCHIN data: limb/extremity/joint arthritic disorders, back, urogenital/pelvic/menstrual, musculoskeletal chest, headache, neck, and multiple pain conditions (Appendix 1) [13]. Variables of interest included demographics, insurance type, encounter and past medical history diagnoses, and prescription information. Persons residing outside of the United States or institutionalized were excluded. 

To identify the medication class of interest (benzodiazepines and opioids), the DailyMed and RxNorm (National Library of Medicine, Bethesda, Maryland, United States) drug class was searched to compile a list of medications in these categories. The OCHIN dataset contained the national drug code (NDC) for patient-prescribed medications. These were cross-referenced to identify medications of interest. MME calculations were performed using conversion factors from the New York State Health Department MME conversion table, which lists the nine-digit NDC, strength per unit, and MME conversion factor for over 2,600 opioids [14].

Description of subset used for this analysis

Patients were divided into two groups: those who received a prescription for naloxone and those who did not. Patients who received a naloxone prescription were grouped into one of five exclusive categories as to potential reasons for the naloxone prescription: (1) concomitant prescription for benzodiazepines (included in high-risk definition), (2) history of OUD (included in high-risk definition), (3) prescribed average daily dose of opioids >50 MME (included in high-risk definition), (4) any combination of the previous three risk factors (included in high-risk definition), and (5) none of the above-described risk factors.

Patients in the benzodiazepine group were prescribed a benzodiazepine 120 days prior or 120 days after receiving an opioid prescription. The criteria for the greater than 50 MME group was an average daily prescription of >50 MME as calculated using the New York State MME conversion table [14]. Patients with a diagnosis of OUD were defined by documentation of OUD prior to the start of their identified opioid prescription episode. Patients in the multiple group fell into two or more of the above definitions. Patients in none group did not fit into the other described groups. 

The primary outcome variable was the prescription of naloxone. Descriptive analysis was done across patient demographics and clinical characteristics to determine whether naloxone prescribing differed between groups. Significant variables were measured using multiple logistic regression and reported using odds ratios, 95% confidence intervals, and p-values. The level of significance used was less than 0.05. The analysis was done using R Version 4.3.2 (R Foundation for Statistical Computing, Vienna, Austria). 

The use of OCHIN data requires adherence to their small cell size suppression policy (Policy124 OCHIN Research) to minimize the likelihood of the identification of individual patients, research data with external partners, and entities for the purpose of protecting patient confidentiality. No cell with patient counts containing a value of 1-10 can be reported or displayed. No cell with patient counts that allows a value of 1-10 to be derived or calculated can be reported or displayed. For any data point that is <10, this is notated as "<10", and any associated values are reported as "> or < X%" to adhere to this patient privacy policy. The study was approved by the Institutional Review Board of the University of Florida as exempt.

## Results

There were 181,964 patients meeting the inclusion criteria. Females represented over 62% (112,703) of the patients, and 53% (96,485) were over 50 years old. Almost half the patients' insurance type was Medicaid (87,870, 48.3%). Among the dataset-defined racial groups, White patients represented 70.9% (129,017) of the cohort, followed by Black/African American (30,406, 16.7%). Hispanic ethnicity was noted among 45,507 patients (25%). Multiple pain conditions (84,409, 46.2%) and back pain (25,407, 14%) were the most common documented pain conditions in the cohort (Table 1).

**Table 1:** Naloxone prescribing demographics p<0.05 set as statistical significance *: value cannot be disclosed due to cell size suppression policy OUD: opioid use disorder; MME: morphine milligram equivalent

N=181,964	Naloxone	No naloxone	P-value
	N=1,807	N=180,157	
Age (years)			
18-20	<10*	2,194 (>90%)*	<0.001
21-30	108 (0.6%)	17,238 (99%)	
31-40	267 (0.9%)	29,086 (99%)	
41-50	322 (0.9%)	36,256 (99%)	
51-60	532 (1.1%)	47,267 (99%)	
61-70	378 (1.2%)	31,204 (99%)	
71-80	147 (1.2%)	12,008 (99%)	
81-90	40 (0.9%)	4,192 (99%)	
91+	<10*	712 (>90%)*	
Race			
American Indian/Alaska Native	22 (1.1%)	2,032 (99%)	0.007
Asian	34 (0.7%)	4,913 (99%)	
Black/African American	351 (1.2%)	30,055 (99%)	
Hawaiian/Pacific Islander	<10*	1,122 (>90%)*	
Multiracial	27 (1.1%)	2,487 (99%)	
Unknown/other	134 (1.1%)	11,761 (99%)	
White	1,230 (1%)	127,787 (99%)	
Hispanic			
No	1,482 (1.1%)	134,975 (99%)	<0.001
Yes	325 (0.7%)	45,182 (99%)	
Sex			
Female	1,066 (0.9%)	111,637 (99%)	0.074
Male	740 (1.1%)	68,471 (99%)	
Other/unknown	<10*	49 (>90%)*	
Payor			
Medicaid	937 (1.1%)	86,933 (99%)	<0.001
Medicare	521 (1.4%)	37,633 (99%)	
Other/unknown	<10*	1,533 (>90%)*	
Private	240 (0.7%)	31,842 (99%)	
Uninsured	101 (0.5%)	22,216 (100%)	
Pain condition cluster			
Back	213 (0.8%)	25,194 (99%)	<0.001
Headache	30 (0.9%)	3,292 (99%)	
Limb/extremity/joint/arthritic	458 (1%)	45,863 (99%)	
Multiracial	1,000 (1%)	94,438 (99%)	
Musculoskeletal chest	32 (1%)	3,329 (99%)	
Neck	52 (1.4%)	3,749 (99%)	
Urogenital/pelvic/menstrual	22 (0.5%)	4,292 (99%)	
History of OUD	273 (7.8%)	3,248 (92%)	<0.001
Benzodiazepines prescribed	468 (2.6%)	17,210 (97%)	<0.001
Average daily MME			
0-24	1,429 (0.9%)	166,157 (99%)	<0.001
25-49	271 (2.4%)	10,799 (98%)	
50-74	63 (3%)	2,009 (97%)	
75-99	26 (4.2%)	600 (96%)	
99+	18 (3%)	592 (97%)	

High-risk features for opioid-related respiratory depression were identified in the population. Among the patients, 3,521 (1.9%) had a history of OUD, 3,308 (1.8%) were prescribed >50 MME per day, and 17,658 (9.7%) were prescribed benzodiazepines (Table 1). 

Of the entire cohort, 1,807 (1%) patients were co-prescribed naloxone. Among patients with high-risk features for OIRD, the group prescribed benzodiazepines (370, 20.5%) were prescribed naloxone more than other defined categories. An additional 1,083 patients received naloxone prescriptions without any of the three personal risk factors of interest documented (Table 2). Examining race among those who received naloxone prescriptions revealed differences: White 1,230 (1%), Black 351 (1.2%), American Indian/Alaskan Native 22 (1.1%), Asian 34 (0.7%), Hawaiian/Pacific Islander <10 (<1%), multiple races 27 (1.1%), and other/unknown 132 (1.1%) (Table 2). Hispanic patients received fewer naloxone prescriptions than non-Hispanic patients (325 (0.7%) vs. 1,482 (1.1%); p <0.001) (Table 1).

**Table 2:** Naloxone prescribing among high-risk characteristics and race All values significant as p<0.05 *: value cannot be disclosed due to cell size suppression policy OUD: opioid use disorder; MME: morphine milligram equivalent

Reason for naloxone	All	American Indian/Alaska Native	Asian	Black/African American	Hawaiian/Pacific Islander	Multiple	Unknown/other	White
N	1,807	22	34	351	<10*	27	134	1,230
Average daily MME ≥50	64 (3.5%)	<10*	<10*	13 (20%)	0 (0%)	<10*	<10*	43 (67%)
Benzodiazepines	370 (20.5%)	<10*	<10*	52 (14%)	<10*	10 (2.7%)	20 (5.4%)	274 (74%)
History of OUD	176 (9.7%)	<10*	0 (0%)	36 (20%)	0 (0%)	<10*	<10*	121 (69%)
Multiple	114 (10.5%)	<10*	<10*	14 (12%)	<10*	0 (0%)	<10*	92 (81%)
None of the above	1083 (59.9%)	<10*	26 (2.4%)	236 (22%)	<10*	11 (1%)	96 (8.9%)	700 (65%)

In the multiple logistic regression model, patients aged 50-80 years were more likely to receive a naloxone prescription than younger and older patients. Those patients receiving >24 MME/day, in particular receipt of 75-99 MME/day, were more likely to be co-prescribed naloxone. Interestingly, patients receiving over 99 MME/day had the lowest OR (OR 1.74; 95% CI 1.07-2.84; p=0.03) of co-prescribing. Medicare (OR 1.31; 95% CI 1.13-1.51; p>0.001) and Medicaid (OR 1.38; 95% CI 1.17-1.63; p>0.001) patients received naloxone more often than private (reference) and uninsured/other/unknown (OR 0.65; 95% CI 0.51-0.82; p>0.001) insurance status. Those with neck (OR 1.57; 95% CI 1.15-2.14; p>0.004), limb/extremity/joint/arthritic (OR 1.23; 95% CI 1.04-1.45; p>0.01), and multiple (OR 1.29; 95% CI 1.11-1.50; p=0.001) pain conditions had a higher likelihood of naloxone receipt. Black/African American patients (OR 1.30; 95% CI 1.15-1.47; p>0.001) were more likely to receive naloxone prescriptions, while Hispanic patients were less likely (OR 0.87; 95% CI 0.76-0.98; p=0.03) (Table 3).

**Table 3:** Logistical regression of naloxone co-prescribing *: p-value significant Bold is used for odds ratios that have a significant p-value (*) CI: confidence interval; OUD: opioid use disorder; MME: morphine milligram equivalent

Characteristic	Odds ratio	95% CI (lower)	95% CI (upper)	P-value
Age (years)				
18-20 (reference)	1	-	-	-
21-30	1.495	0.727	3.073	0.274
31-40	1.96	0.967	3.971	0.062
41-50	1.84	0.909	3.723	0.09
51-60	2.233	1.107	4.504	0.025*
61-70	2.382	1.176	4.828	0.016*
71-80	2.524	1.226	5.198	0.012*
81-90	2.008	0.929	4.339	0.076
91+	1.544	0.499	4.772	0.451
Race				
White (reference)	1	-	-	-
American Indian/Alaska Native	1.05	0.683	1.612	0.825
Asian	0.847	0.599	1.197	0.346
Black/African American	1.297	1.147	1.466	<0.001*
Hawaiian/Pacific Islander	1.011	0.522	1.957	0.975
Multiracial	1.22	0.828	1.797	0.315
Unknown	1.377	1.146	1.655	0.001*
Hispanic				
No (reference)	1	-	-	-
Yes	0.866	0.763	0.983	0.027*
Sex				
Male (reference)	1	-	-	-
Female	0.937	0.85	1.032	0.184
Other/unknown	1.691	0.224	12.756	0.61
Payor				
Private (reference)	1	-	-	-
Medicaid	1.308	1.132	1.511	<0.001*
Medicare	1.379	1.166	1.63	<0.001*
Uninsured/other/unknown	0.646	0.514	0.812	<0.001*
Pain cluster				
Back (reference)	1	-	-	-
Headache	1.292	0.876	1.907	0.196
Limb/extremity/joint/arthritic	1.232	1.044	1.454	0.013*
Multiple	1.286	1.106	1.495	0.001*
Musculoskeletal chest	1.3	0.892	1.895	0.172
Neck	1.571	1.154	2.139	0.004*
Urogenital/pelvic/menstrual	0.869	0.557	1.356	0.537
History of OUD				
No (reference)	1	-	-	-
Yes	6.628	5.757	7.632	<0.001*
Benzodiazepine prescription				
No (reference)	1	-	-	-
Yes	2.763	2.473	3.087	<0.001*
Average daily MME				
0-24 (reference)	1	-	-	-
25-49	2.075	1.81	2.378	<0.001*
50-74	2.236	1.714	2.915	<0.001*
75-99	2.991	1.986	4.506	<0.001*
99+	1.739	1.067	2.835	0.026*

## Discussion

Naloxone is a life-saving medication that can be used to combat OIRD, a potentially fatal condition. In this national cohort, only 1% of patients were co-prescribed naloxone. Among patients with the CDC's high-risk characteristics for opioid-related respiratory depression, those with OUD were more likely to be co-prescribed naloxone, followed by those receiving 75-99 MME/day and patients concomitantly prescribed benzodiazepines and opioids. However, those with the highest MME/day were less likely than lower MME/day groups to receive naloxone prescriptions. Hispanic patients were less likely to be co-prescribed, while Black/African American patients were more likely than other racial groups. The study highlights disparities and underscores the need to adhere more closely to naloxone co-prescribing recommendations among patients in receipt of long-term opioid therapy. 

In 2022, there were close to 15,000 deaths involving prescription opioids [15]. Despite the CDC's recommendations for co-prescribing of naloxone to high-risk patients, studies have shown poor adherence to this potentially life-saving therapy [2,7,16,17]. The study confirms that only a fraction of patients with identified risk factors receive naloxone prescriptions. While previous research has highlighted disparities in naloxone prescribing among Black/African American patients, our findings indicate they are more likely to be prescribed naloxone than White patients, whereas Hispanic patients were less likely. Cost plays a role in access to naloxone among insured and uninsured patients [18,19]. Our study continues to confirm that those who are uninsured are less likely to receive naloxone. To ensure equitable access to the benefits of naloxone, it is crucial to consistently assess risk factors and adhere to precautions for all patients prescribed opioids as well as affordability for all patients.

Efforts to improve naloxone co-prescribing have shown promise to high-risk patients for opioid-related respiratory depression. Green et al. found that states with laws mandating naloxone co-prescribing had a 255% increase after the laws were enacted compared to 90 days prior [9]. As of 2023, 18 states have some laws requiring medical professionals to offer, prescribe, or dispense naloxone. The stipulations vary greatly between states with many thresholds for daily MME use well above the CDC's recommended 50 MME/day [20]. Electronic medical records (EMRs) have proven to be a powerful tool in promoting best practices. Heiman et al. demonstrated that EMR advisories when high-dose opioids are prescribed increased naloxone co-prescribing, which carried over to opioid prescriptions independent of the EMR alert [21]. Given that these interventions are universal to all patient demographics, these are methods to adhere to naloxone prescribing among high-risk patients. Thus, disparities in naloxone prescribing could be reduced.

When examining healthcare databases retrospectively, inherent limitations exist. Granular encounter data, including provider-derived risk stratification, counseling, and offering naloxone but not prescribing, are outside the scope of this dataset. Naloxone obtained from other sources may not be captured. The study ended prior to naloxone becoming available OTC in 2023, alleviating this as a means of obtaining. While the study identifies naloxone prescriptions, the rate of receipt of the actual medication from the pharmacy or insurance coverage benefit for naloxone is unclear. Further studies are needed to confirm dispensing as the scope of this study was to examine the co-prescribing naloxone rate. However, introduction of the benefits of naloxone should be provided by the opioid prescriber. Local regulations regarding the co-prescribing of naloxone vary and could impact the prescribing of naloxone. However, given the large national dataset and the use of uniformed CDC-identified risk factors, particularly the 50 MME/day threshold, these limitations are likely mitigated. Socioeconomic factors are an important consideration in this population. Due to a high rate of missing data for the Federal Poverty Level of patients, insurance status was used as a surrogate. Like previous studies, uninsured patients are less likely to receive a naloxone prescription. It is unclear if prescribers take the cost into account when deciding to co-prescribe. Some naloxone prescriptions were issued to patients without the defined risk factors of interest. When expanding risk factors to include a history of overdose, sleep-disordered breathing and tapering/abstinence with return to high-dose opioids were unable to be identified in the study population [22].

## Conclusions

Patients receiving long-term opioid therapy are at risk for opioid-related respiratory depression that can be lethal. Naloxone co-prescribing can mitigate these adverse outcomes. Our study indicates that patients with identified risk factors are more likely to receive naloxone prescriptions, but there are still significant opportunities to increase its prescribing given its proven life-saving effects and well-established co-prescribing recommendations. Moreover, there continues to be considerable variation in prescribing practices across different populations. To reduce opioid-related morbidity and mortality, robust education and adherence to universal co-prescribing for high-risk individuals are essential. 

## Appendices

Appendix 1

**Table 4:** Additional ICD-10-CM crosswalk search used ICD: International Classification of Diseases Reference: [12]

ICD-10-CM crosswalk search	
Alcohol abuse/dependence	F10%, G62.1, K70%
Opioid abuse/dependence	F11%
Other drug abuse/dependence	F12%, F13%, F14%, F15%, F16%, F18%, F19%
Mood disorders	F06.4, F32.0, F32.1, F32.2, F32.3, F32.4, F32.5, F32.9, F33.0, F33.1, F33.2, F33.3, F33.40, F33.41, F33.42, F33.9, F34.1, F40.00, F40.01, F40.02, F40.10, F40.11, F40.210, F40.218, F40.220, F40.228, F40.230, F40.231, F40.232, F40.233, F40.240, F40.241, F40.242, F40.243, F40.248, F40.290, F40.291, F40.298, F40.8, F40.9, F41.0, F41.1, F41.3, F41.8, F41.9, F42, F43.0, F43.10, F43.11, F43.12, F43.21, F48.8, F48.9, R45.2, R45.5, R45.6, R45.7
Additional pain condition codes	S43.4, G56.2, S22, S81-S89, S91.X-S99.X, S12, S42, S43, S44, S46, S47, S49, S51, S52, S32, T20-T32, z89, T87, S58, S48, S68
